# Gradience Free Nanoinsertion of Fe_3_O_4_ into Wood for Enhanced Hydrovoltaic Energy Harvesting

**DOI:** 10.1021/acssuschemeng.3c01649

**Published:** 2023-07-13

**Authors:** Ying Gao, Xuan Yang, Jonas Garemark, Richard T. Olsson, Hongqi Dai, Farsa Ram, Yuanyuan Li

**Affiliations:** † Co-Innovation Center of Efficient Processing and Utilization of Forest Resources, Nanjing Forestry University, Nanjing 210037, China; ‡ Wallenberg Wood Science Center, Department of Fibre and Polymer Technology, KTH Royal Institute of Technology, Stockholm SE-10044, Sweden; § Key Laboratory of Biomass Chemical Engineering of Ministry of Education, College of Chemical and Biological Engineering, Zhejiang University, Hangzhou 310027, P. R. China; ∥ Institute of Zhejiang University—Quzhou, Quzhou 324000, P. R. China

**Keywords:** Fe_3_O_4_/wood nanocomposites, solvent assisted infiltration, gradience free, hydrovoltaic energy harvesting, water evaporation

## Abstract

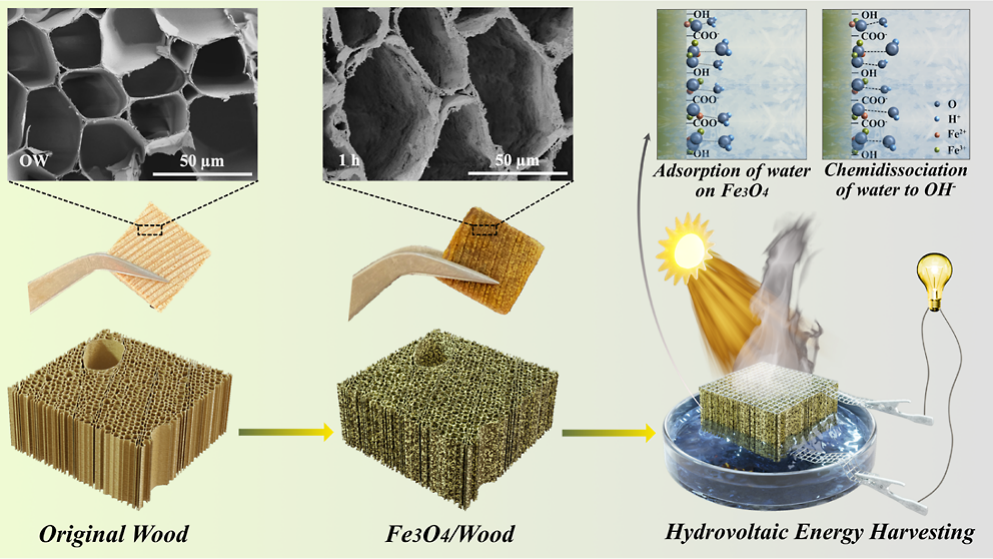

Hydrovoltaic energy harvesting offers the potential to utilize enormous water energy for sustainable energy systems. Here, we report the utilization and tailoring of an intrinsic anisotropic 3D continuous microchannel structure from native wood for efficient hydrovoltaic energy harvesting by Fe_3_O_4_ nanoparticle insertion. Acetone-assisted precursor infiltration ensures the homogenous distribution of Fe ions for gradience-free Fe_3_O_4_ nanoparticle formation in wood. The Fe_3_O_4_/wood nanocomposites result in an open-circuit voltage of 63 mV and a power density of ∼52 μW/m^2^ (∼165 times higher than the original wood) under ambient conditions. The output voltage and power density are further increased to 1 V and ∼743 μW/m^2^ under 3 suns solar irradiation. The enhancement could be attributed to the increase of surface charge, nanoporosity, and photothermal effect from Fe_3_O_4_. The device exhibits a stable voltage of ∼1 V for 30 h (3 cycles of 10 h) showing good long-term stability. The methodology offers the potential for hierarchical organic–inorganic nanocomposite design for scalable and efficient ambient energy harvesting.

## Abstract 2

The current success inspires the potential for hierarchical organic−inorganic nanocomposite design for scalable and efficient ambient energy harvesting. Fe_3_O_4_ nanoparticles were inserted into the wood cell wall without gradience through an in situ synthesis. Acetone-assisted precursor infiltration enabled uniform Fe_3_O_4_ formation in the bulk wood structure. An open-circuit voltage up to 1 V and a power density of ∼743 μW/m^2^ were obtained under 3 suns solar irradiation using Fe_3_O_4_/wood. Enhanced hydrovoltaic energy harvesting is due to increased surface charge, nanoporosity, and photothermal effect from Fe_3_O_4_.

## Introduction

Water is the largest energy carrier on earth, containing around 35% of solar energy received by the Earth, corresponding to over 2000 times the world energy demand.^1^ Renewable energy harvesting from water using nanomaterials and nanotechnologies has attracted wide attention.^2−5^ Hydrovoltaic energy harvesting, water evaporation-induced electricity generation in particular, is promising owing to the considerable energy embedded in the universal evaporation process (around 2/3 of water energy),^6^ applicability to a wide range of materials (such as carbon nanomaterials,^7,8^ Ni–Al alloy,^3,9^ polymers,^10^ and metal–organic frameworks^11^), and easy device fabrication where the electrodes can be used multiple times.^2,7,12^ Electricity generation relies on the interactions between water and materials, mainly based on the classical streaming potential,^13,14^ which is electrical potential generation by driving water through a narrow channel under pressure gradience. Power generation is strongly influenced by the materials’ surface chemistry, which governs the interfacial charging/discharging,^8,11,15^ materials’ nanoporous structure, and water evaporation rate.^8^ Strategies to improve power generation include maximizing the contact area with water, improving the charge separation ability, boosting the water evaporation rate, and so on. Therefore, materials with high nanoporosity, good water wettability, and desirable transportation properties are needed, where three-dimensional structures are highlighted due to increased areal capacity. In addition, green renewable material resources and large-scale processing are favorable for further technological development.

Wood, in this regard, is attractive for hydrovoltaic energy harvesting owing to its renewable origin and hierarchical porous structures, known for efficient water transport through transpiration. Furthermore, wood consists of cellulose, hemicellulose, and lignin, which provide rich functionalities of hydroxyl, carbonyl, and phenolic groups making the wood structure hydrophilic with a negative surface charge.^16^ When water passes through the wood substrate, charge flows inside microchannels resulting in an electrical potential difference along the pressure difference direction. In 2020, native wood was first reported for hydrovoltaic energy harvesting.^14^ Citric acid treatment was applied to improve the energy output. An open circuit voltage (*V*_oc_) of 0.3 V and short circuit current density (*J*_sc_) of 0.4 μA/cm^2^ were achieved. Solar-assisted water evaporation was further reported by coating the wood surface with carbon nanotubes, resulting in a *V*_oc_ of 0.25 V, an *I*_sc_ of 0.48 μA, and a power density of 350 μW/m^2^.^17^ Conductive carbonized wood with abundant hydroxyl groups was also investigated with a *V*_oc_ of 96 mV, an *I*_sc_ of 10.5 μA, and a power density of 294 μW/m^2^.^18^ The challenges lie in the high energy intensive carbonization processing as well as low power density. Recently, our group reported hydrovoltaic energy harvesting from charged nanoporous wood aerogel in a water reservoir at a pH of 13.4. A *V*_oc_ of 0.55 V and power density of 620 μW/m^2^ were obtained under ambient conditions,^19^ indicating the potential of wood nanostructure control for enhanced power generation. Further tailoring nanoengineered wood pH resulted in a *V*_oc_ of 1 V.

Current reports of wood for water evaporation-induced power generation were based on lignocellulose or carbon (carbonized wood) interfacial interactions with water, which shows limited water charge separation abilities. Minerals, such as ZnO and Fe_3_O_4_, are attractive in this context, which have been intensively studied for water splitting.^20^ Among the minerals, iron oxides (e.g., Fe_3_O_4_) are advantageous with additional photothermal abilities,^21^ beneficial for efficient water evaporation, further contributing to enhanced power generation. Formation of a hierarchical nanostructure of iron oxides is attractive yet challenging. We hypothesize that combining iron oxides with a hierarchical structure of wood could provide the possibility to solve the issues and achieve efficient hydrovoltaic power generation.

**Figure 1:**
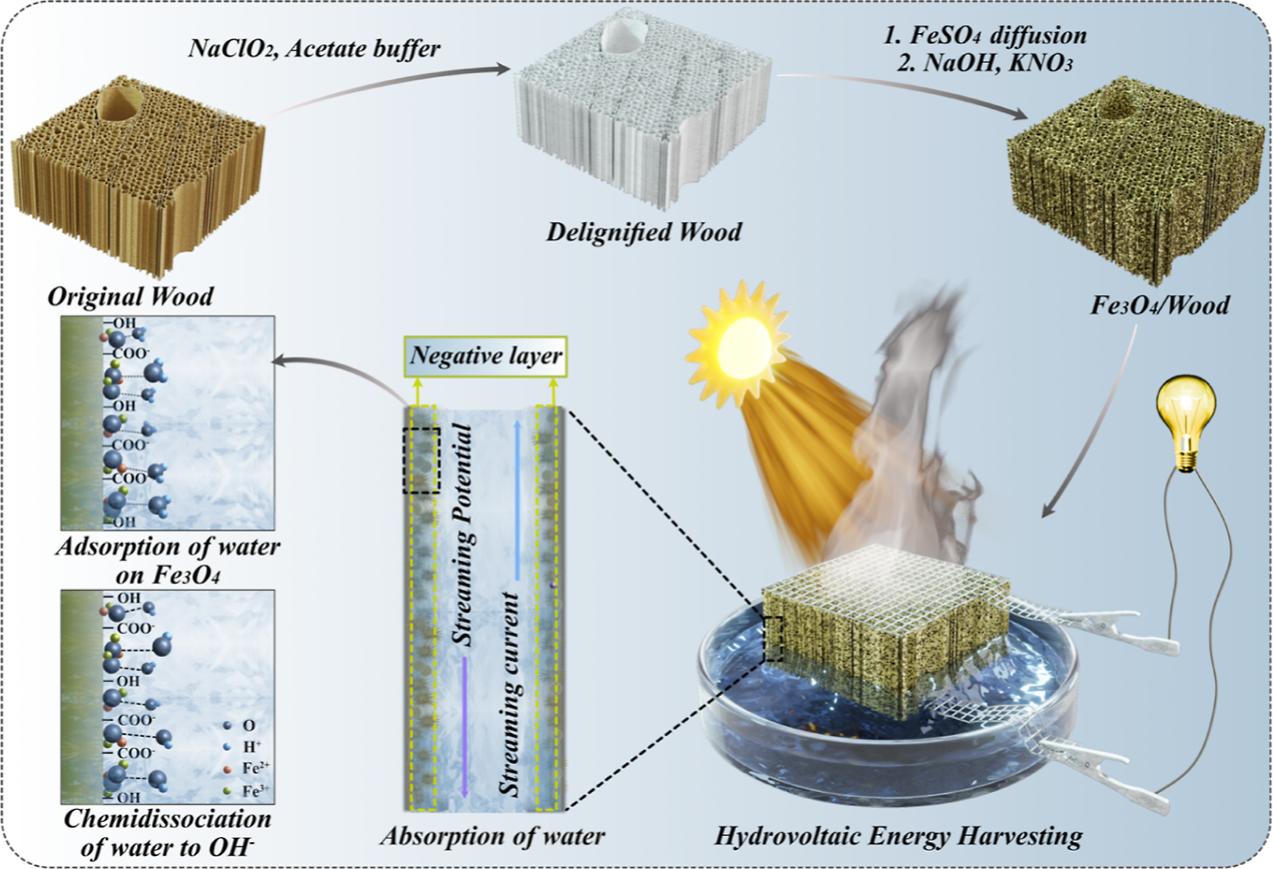
Schematic representation of Fe_3_O_4_/wood (FW) synthesis and the illustration of solar-driven hydrovoltaic energy harvesting.

Direct iron oxide nanoparticle infiltration is one approach, where particle aggregation and diffusion are considerable challenges. In situ nanoparticle synthesis is an alternative with focuses on magnetic functions or photostability.^22−25^ The iron oxide nanoparticle distribution is often limited to the surface regions (wood surface or lumen surface) and gradience issues exist in bulk iron oxide/wood composites,^26^ which may trigger counter voltage generation leading to decreased performance. This hinders the optimum utilization of wood hierarchical structures. In this work, solvent-assisted precursor infiltration in delignified wood is proposed to solve the problems. Figure 1 shows the schematic for hierarchical Fe_3_O_4_/wood nanocomposite formation and further for solar-driven hydrovoltaic energy harvesting. Homogenous coverage of Fe_3_O_4_ nanoparticles both on the lumen surface and in the cell wall was achieved. The Fe_3_O_4_/wood nanocomposite showed improved power generation with a *V*_oc_ of 63 mV and a power density of 51.47 μW/m^2^ under ambient conditions. Under 3 sun irradiation (3 kW/m^2^), a steady *V*_oc_ of 1 V and a power density of 742.66 μW/m^2^ were achieved. This study demonstrates the possibility of wood mineral composites for efficient solar-to-vapor-assisted electricity generation.

## Experimental Section

### Wood Delignification

Original wood (OW) substrates from balsa wood (*Ochroma pyramidale*, purchased from Wentzels Co. Ltd., Sweden) were cut into 1.5 × 1.5 × 1 cm^3^ (tangential × radial × axial) cubes, which have a density of around 110 g/cm^3^. Following our previous methods,^27^ OW cubes were delignified in 1 wt % sodium chlorite (NaClO_2_, Sigma-Aldrich, Sweden) solution with acetate buffer, which controlled pH at 4.6 under 80 °C until the samples became completely white, while the lignin content decreased from 24.9 to 2.9%. After washing several times, all the samples were solvent exchanged and kept in acetone (Sigma-Aldrich, Sweden).

### In situ Growth of Fe_3_O_4_ Nanoparticles

Never-dried delignified wood (DW) was transferred from acetone to 100 mL iron(II) sulfate heptahydrate solution (FeSO_4_.7H_2_O, Sigma-Aldrich, Sweden, 0.1 mol/L) for 1 h under vacuum. The vacuum infiltration could ensure a homogenous distribution throughout the whole wood. Then, the system was heated to 90 °C for 3 h. After thermal precipitation, the samples were transferred into 200 mL 1.32 M sodium hydroxide solution (NaOH, Sigma-Aldrich, Sweden) with potassium nitrate (KNO_3_, Sigma-Aldrich, Sweden) ([Fe^2+^]/[NO_3_^–^] = 0.44), which was heated up to 90 °C. The color of the wood immediately changed from red/orange to dark green/black as nanoparticles formed. After reacting for 10 min, 20 min, 1 h, 2 h, and 4 h, the Fe_3_O_4_/wood (FW) was washed with deionized water (DIW) thoroughly to remove free-moving nanoparticles and ions. Fe_3_O_4_/wood with growth times of 10 min, 20 min, 1 h, 2 h, and 4 h are labeled FW-10 min, FW-20 min, FW-1 h, FW-2 h, and FW-4 h respectively.

### Hydrovoltaic Energy Harvesting

The hydrovoltaic energy harvesters were fabricated by sandwiching the DW or FWs between two Pt mesh electrodes (nominal aperture 0.4 mm, wire diameter 0.1 mm, and 20 × 20 mm^2^), where the electrodes were held together using a cotton thread.^28^ The device was placed in a Petri dish and 25 mL of DI water was added, where the water level was up to 2–3 mm the height of wood to ensure efficient water infiltration in the microchannels. Prior to the device fabrication, the samples were washed using DIW (conductivity of 0.3–0.4 μS/cm) to remove any free ions or Fe_3_O_4_ nanoparticles. Finally, the devices were placed in a Petri dish, and DI water was poured slowly. The open-circuit potential and short circuit current from the wood nanogenerators were recorded in real-time using an electrochemical workstation (CHI Instruments, model 660E), and Keithley's DMM 7510 multimeter at 25 °C and 30% relative humidity. The measurements were carried out by controlling the software on a desktop, and the sampling rate of our instrument was 10 s^–1^. The light was produced by a simulated solar spectrum from a CEL-HXF300-T3 solar simulator (3 kW/m^2^). The surface temperature of the samples was recorded by an infrared camera (FOTRIC 599, China). The total consumption of water was measured after the completion of the experiment and used to calculate the effective rate of water consumption by subtracting the rate of water consumption in an open Petri dish from the rate of water consumption with the sample. The stability of the device is characterized by three cycle tests, each with a duration of 10 h, for a total of 30 h. The output power of a device is dependent on the internal resistance of the device and when the internal resistance is not known, usually, power density is calculated using the external resistors. With the variation of the resistor values (resistance) in the circuit, one can know the maximum power from the device. The maximum power is achieved when the internal resistance of the device matches external circuits. Thus, maximum power can be drawn from a device for practical applications, and to know the maximum power density we have used an external resistor in our work. In this way, power density is calculated using the formula = V*I = *I*^2^*R* = *V*^2^/*R* and divided by area.^29^

### Field Emission-Scanning Electron Microscopy

The wood morphologies were observed by a field-emission scanning electron microscope (Hitachi S-4800, Japan) after sputtering a 10 nm Pt/Pd conductive layer with a sputter-coater (Cressington 208HR, UK). All the images were observed at an acceleration voltage of 3 kV.

### Adsorption Speed Test

The time-dependent infiltration of FeSO_4_ into DW with and without acetone-assisted diffusion was tested by using DW strips (1.0 × 0.1 × 10 cm^3^, tangential × radial × axial). After solvent exchange by using DIW and acetone, the samples were clamped and immersed into 0.1 M FeSO_4_ solution for 1 cm length. The FeSO_4_ content was converted to iron oxide for comparing the loading using thermogravimetric analysis (TGA) under an oxygen atmosphere.

### TGA Measurement

All the diffusion lengths were measured in real-time. TGA (Mettler Toledo-TGA/DSC 1, USA) was performed to measure the composition at a heating rate of 10 °C/min from room temperature to 800 °C under an O_2_ flow of 50 mL/min.

### X-ray Diffraction Analysis

X-ray diffraction (XRD) analysis was carried out by an ARL X’TRA powder diffractometer (Thermo Fisher Scientific Inc., USA) using Cu Kα radiation generated at 45 kV and 44 mA. Scans were obtained from 10 to 80° 2θ in 0.02° steps for 1 s per step. To analyze the influence of growth time on Fe_3_O_4_ crystallite sizes, the average grain size of Fe_3_O_4_ was estimated by Scherrer’s eq 1(30)
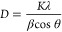
1where *D* is the average diameter of Fe_3_O_4_ crystallites, λ represents the X-ray wavelength (0.154 nm), *K* refers to the Scherrer constant (0.89), β is the full width of the peak at half maximum (full width at half-maximum), and θ represents the Bragg diffraction angle. The average diameters of Fe_3_O_4_ of different FWs were calculated using the characteristic peaks (311).

### Compression Test

The compressive test was performed on a universal testing machine (Instron 5944, UK) under ambient conditions (25 °C and 50% RH). All the measurements were performed using 1.5 × 1.5 × 1 cm (tangential × radial × axial) samples, with a 500 N load cell at a strain rate of 1%/min. The mean value and standard deviation of the modulus for the DW and FW were obtained from the three samples.

### Specific Surface Area Test

The Brunauer–Emmett–Teller (BET) specific surface area was evaluated by nitrogen physisorption. Prior to the nitrogen adsorption, 0.1 g of material was degassed at 90 °C for 1 day, followed by the subsequent BET analysis. The analysis was carried out at −196 °C under a relative vapor pressure of 0.05–0.25 with a Micromeritics ASAP 2020. The BET specific surface area was calculated from the attained isotherms.

### Bulk Zeta Potential Analysis

Zeta potential analysis was conducted using a SurPASS Electrokinetic Analyzer (Anton Paar). DW and FW samples were cut into dimensions of 10 × 10 × 2 mm^3^ (axial × tangential × radial) and attached to a cylindrical cell with double-sided adhesive tape. Prior to the measurements, all the samples were conditioned by immersion in DIW to avoid swelling of wood. Measurements were carried out at 200 mbar with N_2_ in a 0.001 M NaCl electrolyte solution at pH 7.3 ± 0.2. The mean value and standard deviation of the zeta potential (ζ) for the DW and FWs were obtained from three independent ramps measured for two samples each.

## Results and Discussion

**Figure 2:**
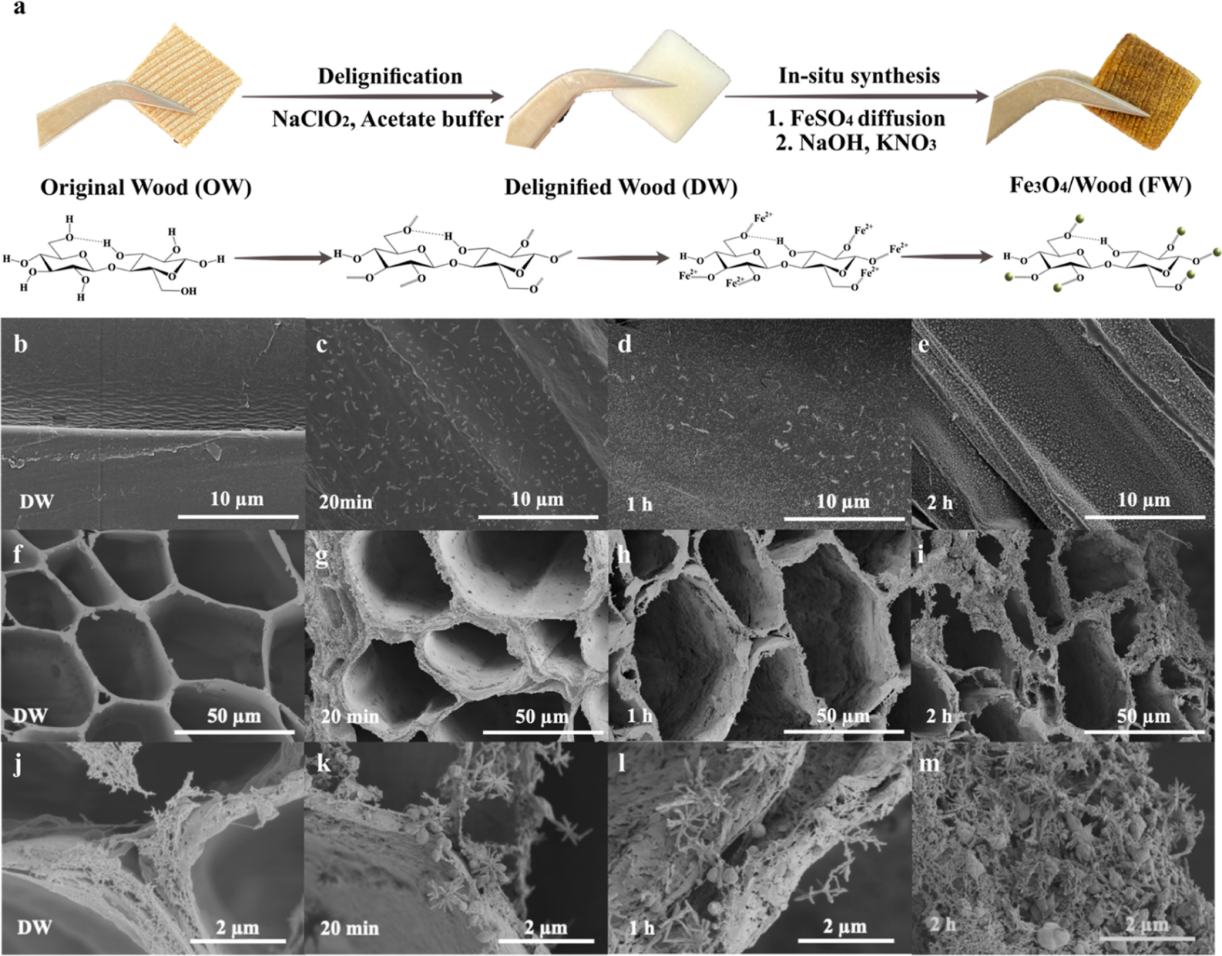
(a) Schematic representation of Fe_3_O_4_/wood (FW) synthesis with a uniform decoration of cell wall by Fe_3_O_4._ Radial face SEM images of DW (b) and FWs with different growth times of 20 min (c), 1 h (d), and 2 h (e). Low magnification and high magnification cross-sectional SEM images of DW (f,j) and FWs with different growth times of 20 min (g,k), 1 h (h,l), and 2 h (i,m).

Figure 2a represents the schematic for the preparation of FW. To facilitate in situ Fe_3_O_4_ synthesis, delignification was first performed to increase the wood structure accessibility.^31^ The removal of lignin from the OW led to a slightly higher porosity (from 92 to 95%) in DW, with an increased BET specific surface area (BET SSA) of 8.7 m^2^/g (1.3 m^2^/g of OW). Meanwhile, a negative surface charge was introduced to the structure due to the oxidation of the C-2, C-3, or C-6 of the monomeric sugar units in polysaccharides, leading to an increased surface charge of 241.3 μeq/g in DW from 25.9 μeq/g of OW. The increased negative surface charge is beneficial for Fe^2+^ adsorption (Figure 2a).^31^ Never dried DW was solvent exchanged to acetone and then infiltrated with FeSO_4_. Thermal precipitation was then applied to promote the transformation of soluble initial iron hydroxides into insoluble iron oxyhydroxide complexes.^32^ Finally, the precipitated precursors inside templates were converted into Fe_3_O_4_ nanoparticles in the NaOH/KNO_3_ solution at 90 °C. The nanoparticle morphology and distribution can be controlled by changing the growth time from 10 min to 4 h, as shown in scanning electron microscopy (SEM) images (Figures 2 and S1).

Figure 2b–m shows the morphology and distribution of Fe_3_O_4_ nanoparticles inside FWs with synthesis time varied from 20 min to 2 h. Compared to DW (Figure 2b), Fe_3_O_4_ nanoparticles started to coat the lumen wall uniformly after 20 min of synthesis (Figure 2c). The nanoparticle cover density improved with increased synthesis time (Figure 2d,e). The cross-sectional images of FWs (Figure 2g–i) revealed the Fe_3_O_4_ layers in the fiber lumens compared to the DW (Figure 2f). Fe_3_O_4_ particles were also present in the region of middle lamella (Figure 2j–m), and even in the cell wall when synthesis time was 2 h. It is noteworthy that harsh alkaline synthetic conditions also gradually destroyed the wood structure, resulting in a loosened-up cell wall structure with increased synthesis time (Figure 2j–m). This porous structure with Fe_3_O_4_ nanoparticle coating could offer a high surface area with enhanced interactions with water,^33^ which is attractive for water evaporation-induced electricity generation. A longer synthesis time of 4 h was further studied, yet the wood structure disintegration started, limiting the further application of the composite (Figure S1f).

**Figure 3:**
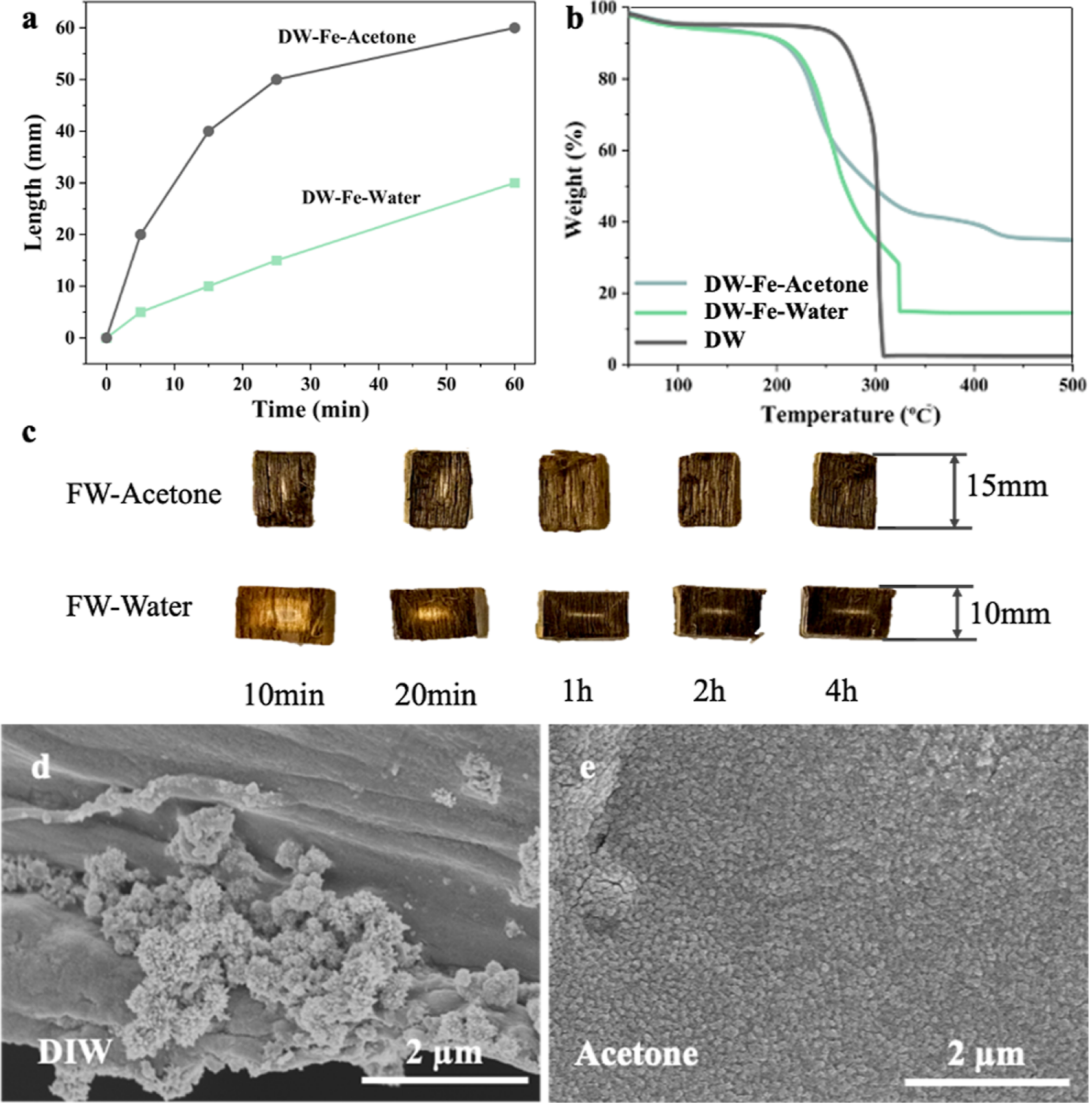
(a) Time-dependent infiltration of FeSO_4_ into DW strips (1.0 × 0.1 × 10 cm^3^ (*T* × *R* × *A*) with and without acetone-assisted diffusion. (b) TGA curves of DW, DW-Fe-water, and DW-Fe-acetone under O_2_. (c) Photos of FW samples (cut from the middle) with (FW-acetone) and without (FW-water) acetone-assisted infiltration. FW-acetone sample size is 1.5 × 1.5 × 1.5 cm^3^ (*T* × *R* × *A*) cubes, and the FW-water sample size is 1.5 × 1.5 × 1 cm^3^ (*T* × *R* × *A*) cubes. *T* is for tangential, *R* is for radial, and *A* is for axial. High-resolution radial face SEM images of Fe_3_O_4_/wood (FW) (d) without acetone-assisted infiltration and (e) in the presence of acetone-assisted infiltration.

Obtaining uniform Fe_3_O_4_ nanoparticle decoration inside the wood is important to the final device performance. The current reported FWs through in situ synthesis always shows an obvious Fe_3_O_4_ nanoparticle gradience. The main issue lies in the chemical diffusion during the synthesis, in particular metal-ion diffusion. To solve the problem, acetone-assisted FeSO_4_ infiltration was applied. Figure 3a shows the time-dependent FeSO_4_ diffusion into the DW, exhibiting faster diffusion in DW that is solvent exchanged to acetone (DW-Fe-acetone). In addition, higher precursor loading is also achieved for the DW-Fe-acetone (Figure 3b). Under an O_2_ atmosphere, the TGA residue of the DW is near 0% due to the burning of cellulosic materials. The iron oxide residue (formed under an O_2_ atmosphere in TGA) for FeSO_4_ infiltrated DW without acetone exchange (DW-Fe-water) is ∼15 wt %, which increased to 35 wt % for DW-Fe-acetone. This indicates a higher amount of FeSO_4_ in the sample. Figure 3c shows the final FW samples after hydrothermal growth with and without acetone-assisted FeSO_4_ infiltration. Although with thicker samples, no obvious Fe_3_O_4_ nanoparticle gradient issue is present for FW-acetone after 1 h of reaction. Without acetone-assisted FeSO_4_ infiltration, FW samples always show a gradient even after a reaction time of 4 h. More importantly, acetone-assisted infiltration ensures a homogenous deposition of nanoparticles, which otherwise are agglomerated in the absence of acetone-assisted infiltration (Figure 3d,e). In addition to acetone, ethanol, and hexane were also studied to assist metal-ion diffusion. The results of the adsorption capacity of iron ions after infiltration (Figure S2) support the best efficiency of using acetone.

The proposed mechanism for enhanced metal-ion diffusion could be described as the following. Acetone is known to be miscible with water in any proportion under ambient conditions and to be able to wet wood completely,^34^ ensuring acetone’s penetration into the wood cell wall and wetting all the surfaces. Therefore, DW was solvent exchanged to acetone before FeSO_4_ infiltration. Osmotic pressure drives the diffusion of acetone from the cell wall into the FeSO_4_ solution, while facilitating the diffusion of FeSO_4_ solution into the wood cell wall. In addition, acetone is highly volatile. Fast evaporation of acetone could enhance the pressure difference, which further contributes to faster metal-ion diffusion in DW. More importantly, acetone infiltration ensures a homogenous deposition of nanoparticles which are agglomerated in the absence of acetone infiltration (Figure 3d,e).

A series of characterizations were performed to evaluate the effect of wood mineralization by Fe_3_O_4_ on hydrovoltaic energy-harvesting performance and to understand the working mechanism. Zeta potential is an important parameter that influences the potential generation. Increased zeta potential was recorded for DW and FWs (Table 1). The Fourier-transform infrared (FTIR) spectroscopic analysis of delignified samples confirmed increased −COO^–^ functionalities indicated by the increased peak intensity at around 1724 cm^–1^ (Figure S2). As a result, the negative zeta potential of the wood bulk substrate increased from −15.4 to −21.2 mV (Table 1). Additionally, these increased functional groups provided more reaction sites for the metal-ion binding, making it easier for the wood substrate to get mineralized. In situ synthesis of Fe_3_O_4_ further increased the zeta potential to −24.2, −37.7, and −32.6 mV in FW-20 min, FW-1 h, and FW-2 h, respectively. The increased zeta potential could be attributed to the ability of Fe_3_O_4_ to chemidissociate water.^21^ The decrease of zeta potential for FW-2 h compared with FW-1 h could be due to the significant removal of hemicellulose, which is the main source of charge in DW during the Fe_3_O_4_ synthesis under alkaline conditions.

**Table 1:** Basic Properties of DW and FW Samples

	DW	FW-20 min	FW-1 h	FW-2 h
zeta potential (mV)	–21.2	–24.2	–37.7	–32.6
BET SSA (m^2^/g)	8.7	1.9	1.6	4.5
Fe_3_O_4_ content (%)		14.4	19.9	22.6
modulus (MPa)	65.4	36.2	32.5	28.3

**Figure 4:**
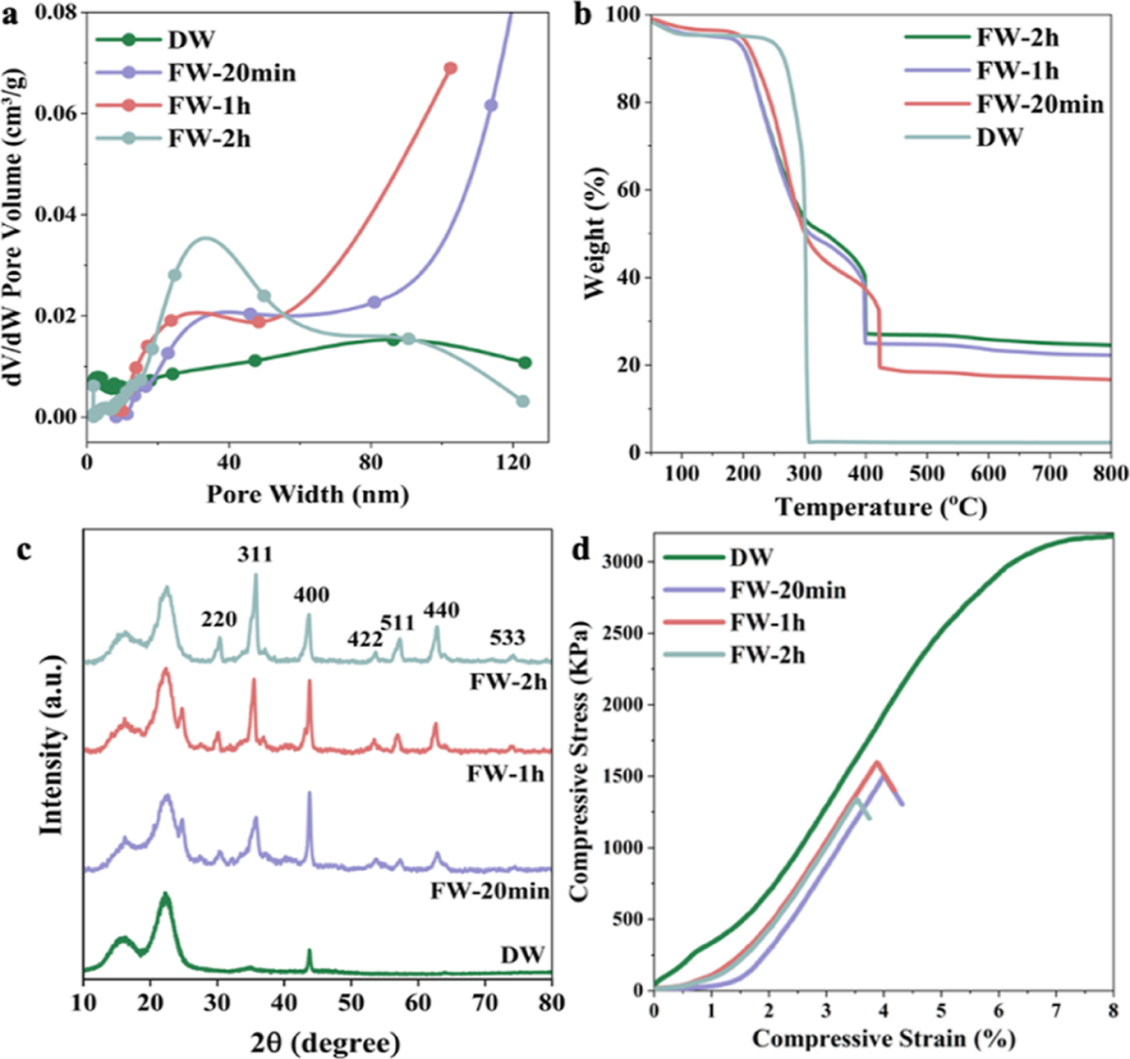
Characterizations of DW and FWs (20 min, 1 h, and 2 h), (a) desorption d*V*/d*W* pore size distribution, (b) TGA curves under O_2_, and (c) XRD patterns, and (d) wet mechanical property.

The SSA and pore size distribution are also greatly affected by Fe_3_O_4_ modifications (Table 1 and Figure 4a). The SSA first decreased from 8.7 m^2^/g (DW) to 1.9 m^2^/g (FW-20 min) and was further followed by an additional decrease to 1.6 m^2^/g (FW-1 h). A slight SSA increase was obtained for FW-2 h with a value of 4.5 m^2^/g. The decreased SSA compared with DW is mainly due to increased Fe_3_O_4_ content, which has higher density compared with wood. TGA showed increased Fe_3_O_4_ contents with increasing reaction time. FW-20 min, FW-1 h, and FW-2 h show particle contents of 14.4, 19.9, and 22.6 wt %, respectively (Figure 4b and Table 1). The SSA increase of FW-2 h compared with FW-1 h could be attributed to the increased mesoporosity. The FW-20 min and FW-1 h showed high porosity with pore sizes ≥120 nm, while FW-2 h exhibited high pore volume with a pore size of around 30 nm. One hypothesis is that the harsh alkaline conditions for Fe_3_O_4_ nanoparticle synthesis destroyed the wood structure by hemicellulose diffusion out from the structure and holocellulose degradation, generating nanoscale pores. Before a reaction time of 1 h, the influence of Fe_3_O_4_ nanoparticles is prevalent. After 2 h growth, the effect of mesopore generation is predominant, leading to increased SSA of FW-2 h although with a higher Fe_3_O_4_ content.

Figure 4c displays the XRD patterns of DW and FWs with different growth times. The XRD patterns show variations at the 002-diffraction peak (2θ = 22.6°), which are associated with the crystalline region of the cellulose. The diffraction peaks of FWs at 2θ of 30.3, 34.4, 35.8, 53.3, 57.3, and 74.3° can be assigned to (220), (311), (422), (511), (440), and (553) crystal planes of Fe_3_O_4_ (JCPDS: 19-0629). A small peak at ∼25° in FW-20 min and FW-1 h could be attributed to Fe_2_O_3_ suggesting the presence of mixed iron oxides (JCPDS: 33-0664). Further, this peak disappeared for FW-2 h (Figure 4c), suggesting better purity of Fe_3_O_4_. Wet mechanical properties are essential for material handling during the device assembly and running. Figure 4d shows the stress-strain curve of samples under compression tests along the longitudinal direction, data is summarized in Table 1. As a reference point, the DW showed a Young’s modulus of 65.4 MPa and a compression strength of 2500 kPa. Although the preparation in a strong alkaline solution resulted in decreased mechanical properties (an elastic modulus of 32.5 MPa and strength of 1650 kPa) for FWs, the samples remain mechanically stable during long-term device usage. Moreover, the ∼160 times enhancement in the power density further mitigates these concerns. The mechanical properties of magnetic wood are an issue and especially because of the use of alkali conditions, and longer treatment led to reduced mechanical properties. However, after 2 h of alkali treatment, the hydrovoltaic energy-harvesting performance also drops and for practical applications, the alkali treatment can be stopped after 2 h. At 2 h of alkali treatment for Fe_3_O_4_ synthesis, FW-2 h (∼1 cm thick) is relatively stable and we have explored it for hydrovoltaic energy harvesting for 30 h without compromising the performance. Also, the literature suggests that the optimum performance of wood-based hydrovoltaic devices is obtained at a thickness of 1 cm, and no further advantages are seen for thicker samples.^14^ Handling a larger sample is a relatively small issue, which can be mitigated by assembling many devices for a practical application.

**Figure 5:**
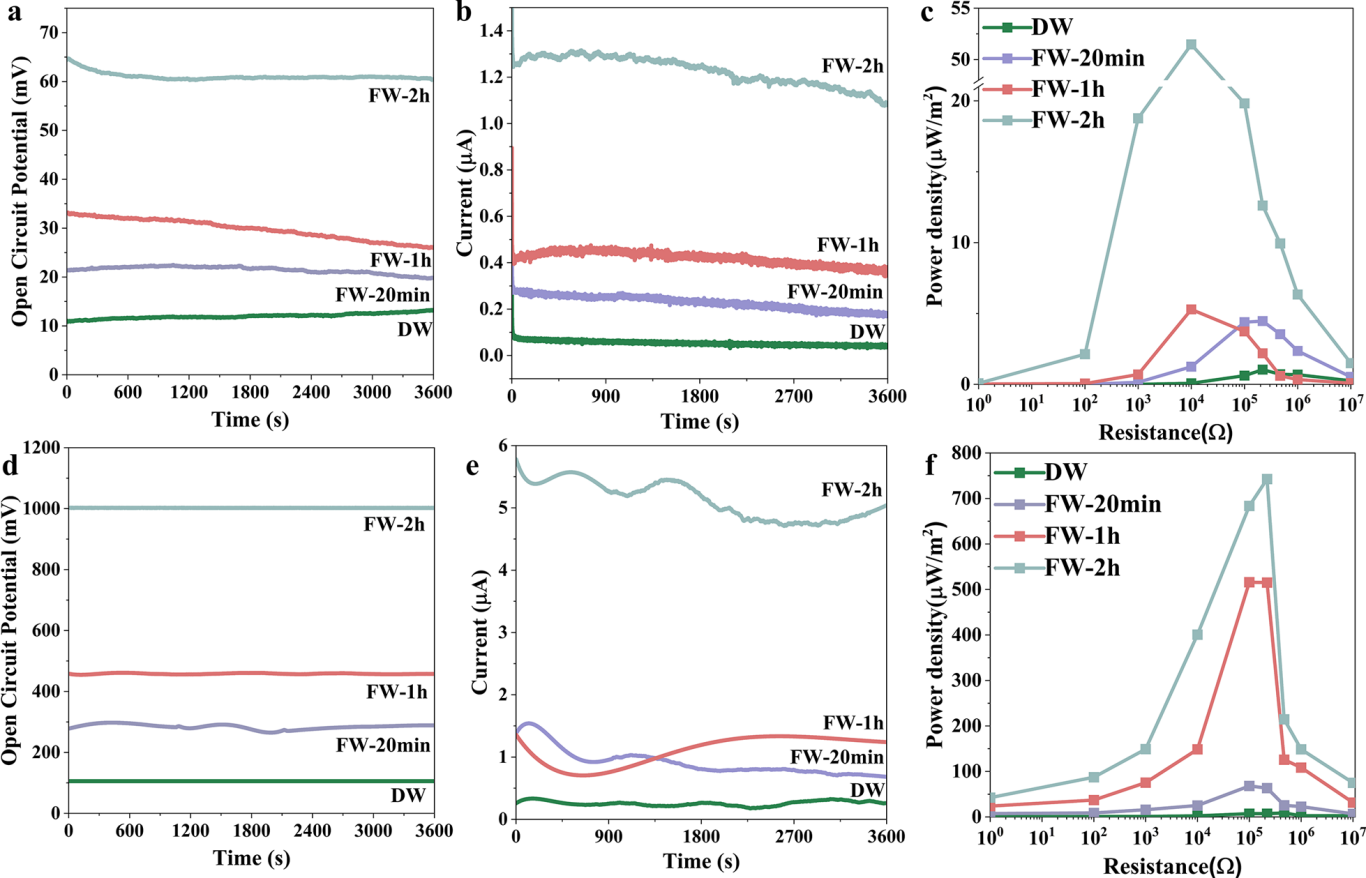
Hydrovoltaic energy harvesting from DW and FWs under ambient conditions: (a) *V*_oc_, (b) *I*_sc_, and (c) power density with the variation of external resistors. Hydrovoltaic energy harvesting from DW and FWs under three sun irradiation: (d) *V*_oc_, (e) *I*_sc_, (f) power density with the variation of external resistors.

The FWs (20 min, 1h, and 2h) were evaluated for hydrovoltaic energy harvesting in terms of *V*_oc_ (Figure 5a), *I*_sc_ (Figure 5b), and power density (Figure 5c). A continuous direct voltage can be harvested from wood from the water flow driven by natural evaporation.^35^ After delignification, *V*_oc_ was slightly improved with a value of 11 mV compared to 9.3 mV from OW. This could be attributed to the enhanced surface charge and SSA. An *I*_sc_ of ∼0.06 μA was obtained for DW. A further increase was noticed for the FWs with dependency on growth time. FW-20 min and FW-1 h resulted in *V*_oc_ of ∼21 and ∼30 mV, while the *I*_sc_ were 0.22 and 0.39 μA, respectively. The FW-2 h rendered the highest *V*_oc_ of ∼63 mV and an *I*_sc_ of ∼1.17 μA. For the FW-20 min and FW-1 h, with comparable SSAs, the output performance was higher for FW-1 h probably due to higher Fe_3_O_4_ content. However, the weight fraction of Fe_3_O_4_ is slightly increased from the FW-1 h to FW-2 h, and the hydrovoltaic energy-harvesting performance is superior to FW-2 h, suggesting that the porosity at the nanoscale has a major effect on the output performance over the weight fractions of Fe_3_O_4_.

The power density was measured by placing the resistors with variable resistance in the circuit (Figure S4). Resistance dependencies on power density are shown in Figure S4. The maximum power density of DW was 1.03 μW/m^2^ under an external load of 2.2 × 10^5^ Ω. Improved power density was observed for FW-20 min with a value of 4.46 μW/m^2^ under the same external load. Further, an improved power density of 5.28 μW/cm^2^ was obtained for FW-1 h. It should be noted that the maximum power density was obtained with an external load of 10^4^ Ω, suggesting a change in the internal resistance of the FW samples. FW-2 h resulted in the highest power density of 51.47 μW/m^2^ at an external load of 10^4^ Ω (Figure 5c), ∼165 times higher than that of OW running in a similar condition (about 0.31 μW/m^2^).

Increasing the water evaporation rate further improves the power output. This is one of the main reasons using Fe_3_O_4_ to decorate wood for hydrovoltaic energy harvesting. The wide solar adsorption and strong photoresponse of Fe_3_O_4_^36^ could contribute to water evaporation through photothermal conversion, which has been intensively investigated.^37^ For all samples, a drastically enhanced performance was observed under 3 sun irradiation compared to that under ambient conditions mainly due to the increased water evaporation rate under higher solar irradiation intensity. As shown in Figure 5d, the *V*_oc_ and *I*_sc_ for DW are ∼105 mV and ∼0.29 μA, respectively. FW-20 min and FW-1 h resulted in *V*_oc_ of ∼285 and ∼457 mV, whereas the *I*_sc_ were ∼0.87 and ∼1.11 μA (Figure 5e) respectively. The *V*_oc_ of FW-2 h reached up to 1 V with an *I*_sc_ of ∼5.17 μA. The power densities also showed a remarkable increase (Figures 5f and S5), where a maximum power density of 8.81 μW/m^2^ was achieved for DW at an external load of 4.7 × 10^5^ Ω. FW-20 min and FW-1 h resulted in the maximum power density of 68.06 and 515.85 μW/m^2^ under 10^5^ Ω load. The FW-2 h resulted in the highest power density of the study, which was found to be 742.66 μW/m^2^ and it was obtained at an external load of 2.2 × 10^5^ Ω. The power density is remarkably increased by 1300% compared to ambient conditions ascribed to the excellent solar photothermal conversion property of Fe_3_O_4_. The performance of FWs under normal and three sun irradiation are comparable, or higher than the previous reports (Table S3).

**Figure 6:**
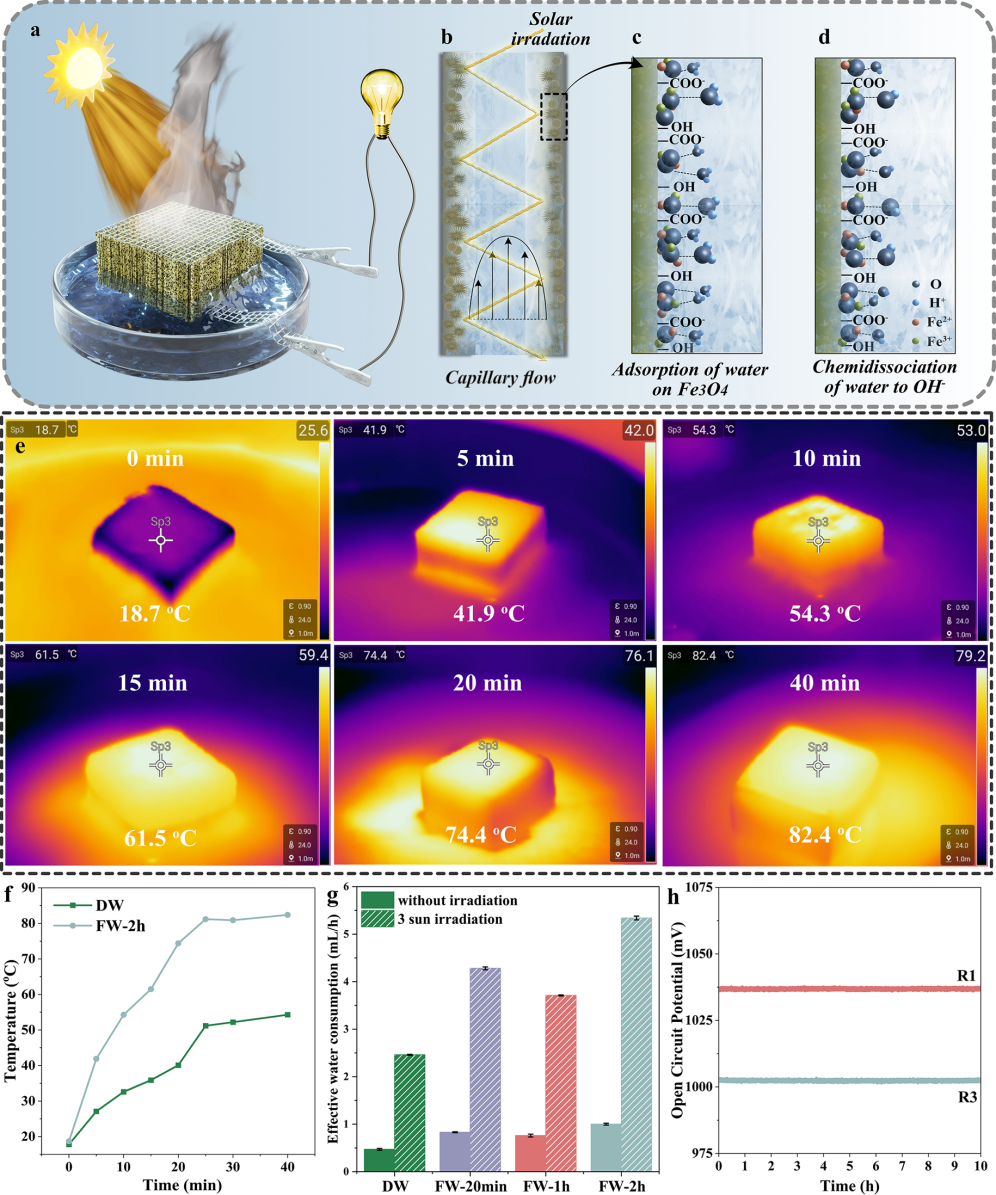
(a) Device structure where two Pt mesh electrodes are attached to the top and bottom of DW or FWs and the microchannels of the wood are vertically aligned. (b) Longitudinal section of wood fiber, where Fe_3_O_4_ nanoparticles are attached to the lumen surface in FWs, whereas they are absent in the DW. The schematic represents the generation of streaming potential across the capillary tube due to water flowing through it, where walls are negatively charged. (c) Adsorption of water molecules on the Fe_3_O_4_ nanoparticles and (d) their chemidissociation in FWs. (e) Heat localization effect of the FW-2 under 3 sun simulated irradiation. (f) Surface temperature of DW and FW-2h under 3 sun irradiation. (g) Rate of effective water consumption without irradiation and under 3 sun irradiation. (h) Long-term stability and cyclic durability of the FW-2h.

Figure 6a shows the device structure and proposed mechanism for hydrovoltaic energy harvesting. When water contacts wood, adsorption of water on the wood surface occurs. Wood has a net negative surface charge due to the presence of hydroxyl groups, carboxylic groups, and Fe_3_O_4_ nanoparticles. An electrical double layer forms from the adsorption of counterions. A pressure difference at the two ends of the wood leads to directional motion of counterions, resulting in an electrical potential difference (Figure 6b). Water evaporation-induced electricity is a new topic with limited understanding of the working mechanisms. Streaming potential,^13,14^ evaporation potential,^38^ ionovoltaic voltage generation,^39^ and so on have been proposed, yet it is commonly agreed that streaming potential is the main contribution. For this specific case, the mechanism is even more complicated as solar irradiation is applied and Fe_3_O_4_ could potentially be a catalyst for reactions under solar irradiation. To elucidate the working mechanism, we combined the following different theories for analysis in the process of interpreting the experimental results. First, in this process, we treat the wood as a bundle of capillaries assembled so that an electric double-layer effect occurs when water contacts the charged surface of the wood. In addition, the cubic inverse spinel structure of Fe_3_O_4_ particles is active for water adsorption,^40,41^ leading to heterogeneous dissociation of water molecules, thereby enhancing the hydrovoltaic energy harvesting capability.^21^ As for the photothermal effect in the process, we mainly believe that it improves the evaporation capacity of water. Other possible contributions were also proposed such as chemical reactions (solar water splitting) in the structure and solvent evaporation potential.

In the system, wood could be considered as bundles of cylindrical capillary tubes with a length of *L*, pore diameter of *d*, and the inner surface zeta potential of ζ. If only streaming potential (*V*_s_) is considered for potential generation, the potential could be described according to the following eq 2
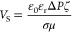
2where ε_0_, ε_r_, σ, and μ are the electrical permittivity of vacuum, the relative permittivity of the solution, the conductivity, and the viscosity of the solution, respectively. Δ*P* is the external pressure difference. When DIW is applied as the reservoir, ε_0_, ε_r_, σ, and μ are constant. External pressure difference (Δ*P*) and wood surface zeta potential (ζ) are critical variables for potential generation.

For FWs, the interaction between water molecules and Fe_3_O_4_ particles contributes to enhanced ζ (Table 1). As shown in schematic in Figures 6a–d, when water enters the wood pores through the capillary force, it will be adsorbed on the surface of Fe_3_O_4_ nanoparticles. This is facilitated by the cubic inverse spinel structure of the Fe_3_O_4_ nanoparticle, which is active toward water adsorption^40,41^ due to the attraction between lone pairs of oxygen in the water molecules and Fe cations (Fe^2+^ and Fe^3+^) (Figure 6c). This decreases the–OH bond dissociation energy resulting in a heterolytic dissociation of water. A new Fe–OH bond forms and the H^+^ ion binds to nearby surface oxygen forming another Fe–OH bonds (Figure 6d).^4^ Hence, the chemidissociation of water on the Fe_3_O_4_ surface increases the inner zeta potential (ζ) by surface hydroxylation for enhanced hydrovoltaic energy harvesting.^21^ There could also be contributions from the thermoelectric effect, solvent evaporating potential^38^ and ionovoltaic voltage generation.^39^ Therefore, eq 2 is not directly applicable to calculate the generated output voltage.

The high performance under solar irradiation could be attributed to the enhanced pressure drop due to the increased water evaporation rate. For identical straight parallel cylindrical capillaries, the pressure drop can be expressed by following eq3^42^
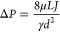
3where *J* is the volume flux (or effective water evaporation rate), and γ is the porosity. The increased water evaporation rate was observed under solar irradiation especially for FWs due to the high photothermal conversion ability of Fe_3_O_4_. The photothermal conversion ability of Fe_3_O_4_ is supported by the higher sample surface temperature under solar irradiation (Figure 6e,f). The surface temperature of FW-2 increased from 18.7 to ∼82.4 °C in 20 min, while only reaching ∼54.3 °C from 17.8 °C for DW (Figure S6a). The rate of total water consumption increased over 5 times under 3 sun irradiation (Figure S6b,c). The effective water consumption under ambient conditions were 0.47, 0.82, 0.76, and 1 mL/h for DW, FW-20 min, FW-1 h, and FW-2 h (Figure 6f), which increased to 2.46, 4.28, 3.71, and 5.34 mL/h, respectively. The trend for effective water consumption rate increase agrees with the potential generation change.

It should be noted that the measured *V*_oc_ is not linearly proportional to pressure drop (Δ*P*)/effective water consumption rate (*J*), and wood surface zeta potential (ζ). This indicates the presence of other important factors that influence the power generation, such as the nanoporosity. Pores at the nanoscale could have a significant effect on the output performance by building an overlap of diffuse parts of electric double layers. The effect could be over the influence of the surface zeta potential. In this work, FW-2 h shows higher output performance than the FW-1 h, even if the latter has a higher zeta potential. This could be assigned to the pore size distribution/porosity difference at the nanoscale, which plays a vital role in hydrovoltaic energy harvesting. As shown in Table 1 and Figure 4a, FW-2 h exhibits high pore volume with a smaller pore size (∼30 nm), while FW-20 min and FW-1 h show mainly pore sizes ≥120 nm. At the same time, the bulk porosity increased for FWs, from 93.6% (FW-20 min) to 93.8% (FW-1 h) and 96.3% (FW-2 h). This indicates that higher SSA/porosity at the nanoscale maximizes the interaction between the charged surface and water for enhanced performance. As discussed earlier, in addition to streaming potential, other mechanisms may also be involved and further efforts are needed to understand the working mechanisms.

With the incorporation of Fe_3_O_4_ nanoparticles, FWs not only show higher power generation, which could be attributed to the increased surface charge, nanoporosity, and photothermal effect from Fe_3_O_4_, but also good stability. The device performance stability was estimated by running the samples multiple times. After 3 cycles (equivalent to 30 h), the FW-2 h retained the 96.7% *V*_oc_ generated (Figure 6h). This suggests that wood mineralization can offer a rational design for hydrovoltaic energy harvesting using organic–inorganic nanocomposites for sustainable energy harvesting from the environment. The individual hydrovoltaic nanogenerator has very low power. The DW and FW-2 h can also be used under real outdoor conditions; here, we tested the output voltage under different temperatures and pH (Figures S7 and S8). However, with the assembly of many similar devices, the power output can be enhanced, which can run small electronics, such as calculators, watches, and so on. The key advantage of these devices is continuous power generation for a longer time, which can also be stored in a capacitor for later use.

## Conclusions

In conclusion, Fe_3_O_4_ nanoparticles were successfully incorporated into a hierarchical wood structure for hydrovoltaic energy harvesting through an in situ synthesis. Acetone-assisted precursor infiltration ensures nanoparticles’ uniform distribution throughout the wood structure and into the cell wall. A *V*_oc_ of 63 mV, *I*_sc_ of 1.17 μA, and power density of ∼52 μW/m^2^ were obtained for FW-2 h, which is over 165 times higher compared with the OW. The enhanced performance was ascribed to the higher surface charge, the nanoporosity, and the water dissociation ability of Fe_3_O_4_ under ambient conditions. By further enhancing the evaporation rate by using solar energy, a *V*_oc_ of 1 V and the highest power density of 742.66 μW/m^2^ were obtained, which was mainly due to the enhanced rate of effective water consumption and the photothermal effect of Fe_3_O_4_. The current success inspires further investigation of other mineralized wood for energy harvesting under solar irradiation through a careful structure design. The assembled wood-based devices can be used for continuous power generation over long periods of time.

## Supporting Information Available

Experimental results concerning FTIR spectroscopy spectra of OW and DW and hydrovoltatic energy harvesting from DW and FW-20 under different temperatures and pH (PDF)
