# Supplementary material for: Gradience Free Nanoinsertion of Fe3O4 into Wood for Enhanced Hydrovoltaic Energy Harvesting
Source: ACS Sustain Chem Eng. 2023 Jul 13;11(30):11099–109. doi: 10.1021/acssuschemeng.3c01649 (PMC10394687; doi:10.1021/acssuschemeng.3c01649)
Supplement: Supplementary file 1 — sc3c01649_si_001.pdf [file sc3c01649_si_001.pdf]

Supporting Information

Authors: Ying Gao[1,2], Xuan Yang[2,3,4], Jonas Garemark[2], Richard T. Olsson[2], Hongqi Dai[1], Farsa Ram[2,5*] and Yuanyuan Li[2*]

[1]Co-Innovation Center of Efficient Processing and Utilization of Forest Resources, Nanjing Forestry University, Nanjing 210037, China

[2]Wallenberg Wood Science Center, Department of Fibre and Polymer Technology, KTH Royal Institute of Technology, SE-10044 Stockholm, Sweden

[3]Key Laboratory of Biomass Chemical Engineering of Ministry of Education, College of Chemical and Biological Engineering, Zhejiang University, Hangzhou 310027, P.R. China

[4]Institute of Zhejiang University-Quzhou, Quzhou 324000, P.R. China

[5]Pritzker School of Molecular Engineering, University of Chicago, 5640 South Ellis Avenue, Chicago, Illinois 60637, United States

* Corresponding Authors:

Farsa Ram: farsa@kth.se

Yuanyuan Li: yua@kth.se

Manuscript title: Gradience free nano insertion of Fe_3O_4 into wood for enhanced hydrovoltaic energy harvesting

The number of pages: 11

The number of figures: 8

The number of tables: 4

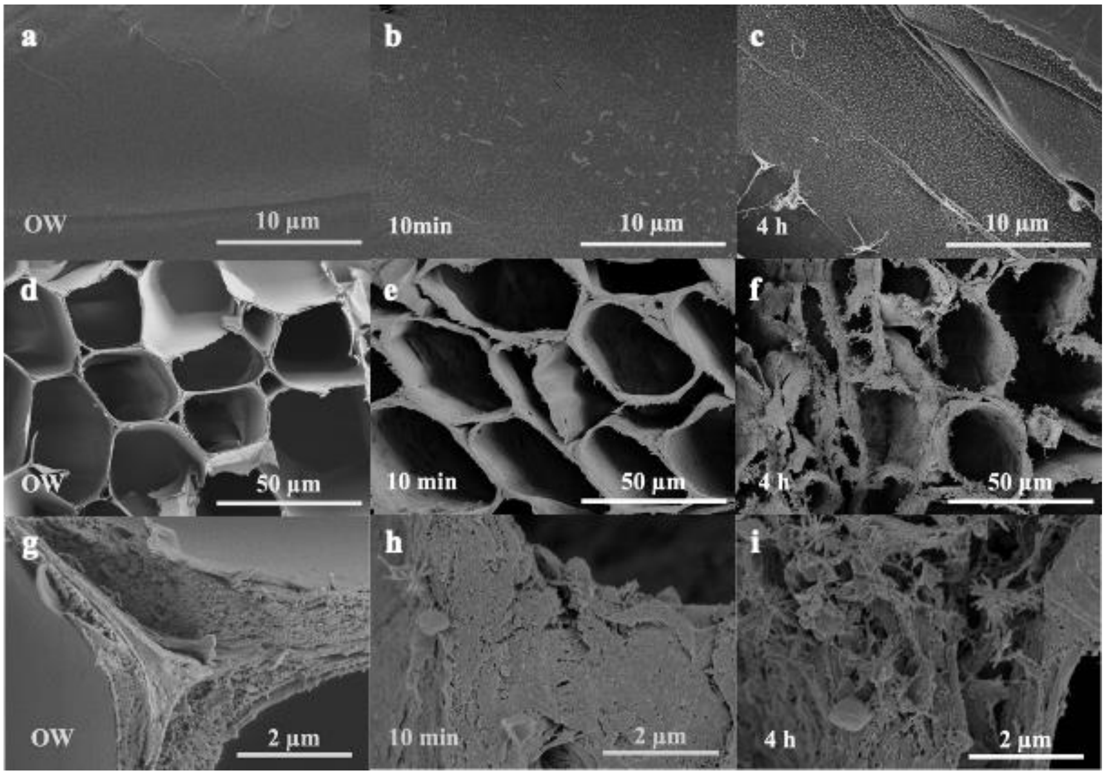

Figure S1. Radial face SEM images of OW (**a**) and FWs with different growth times, 10 min (**b**) and 4 h (**c**). Low magnification and high magnification cross-sectional SEM images of OW (**d** and **g**) and FWs with different growth times, 10 min (**e** and **h**) and 4 h (**f** and **i**).

Fourier-transform infrared spectroscopy (FTIR) spectra were obtained at 16 scans and a resolution of 4 cm^{-1} in transmission mode with a Spectrum 100 FT-IR Spectrometer (Perkin Elmer, UK). The scan range was fixed between 600 and 4000 cm^{-1}. During the sodium chlorite ($NaClO_2$) delignification step, the lignin aromatic structure undergoes an oxidative ring-opening reaction to form acidic groups, while the C-2, C-3, or C-6 on monomeric sugar units were oxidized to carbonyl (-C=O) or carboxylic groups (-COO^-)[1]. 1600 cm^{-1} peak is assigned to lignin and 1730 cm^{-1} peak is assigned to carboxylic groups.

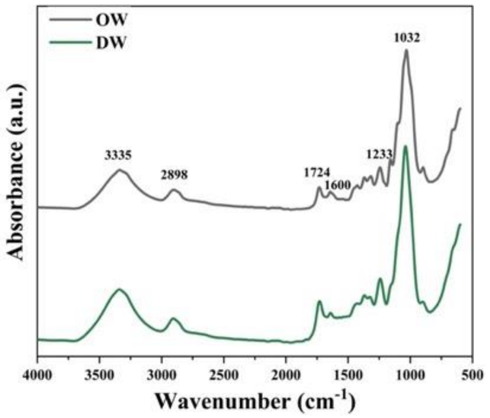

Figure S2. FTIR spectra of OW and DW.

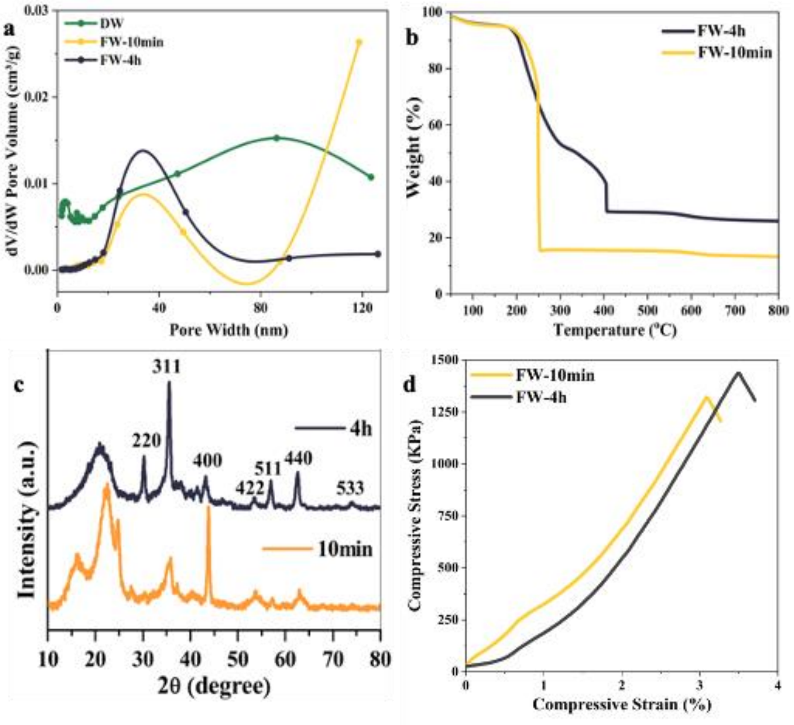

Figure S3. a Desorption dV/dlog(w) pore size distribution of DW and FW (10min and 4h). **b** TGA curves (under O_2) of DW and FW (10min and 4h). **c** XRD patterns of FW (10min and 4h). **d** Compressive stress-strain curves of DW and FW.

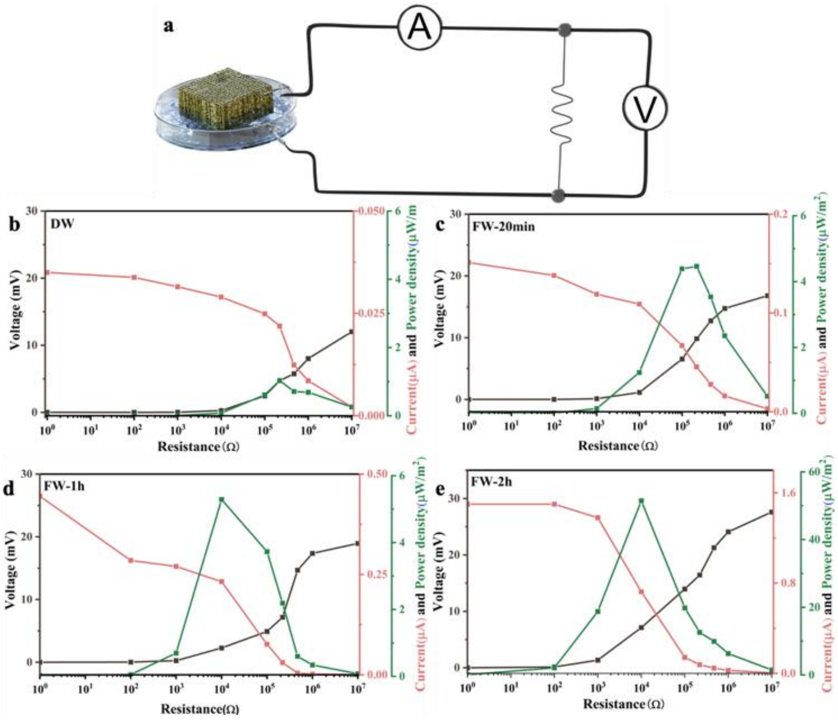

Figure S4. The testing electrical circuit (**a**) and resistance dependence on voltage, current and power density of DW (**b**) and FWs with different growth times 20 min (**c**), 1h (**d**), and 2h (**e**).

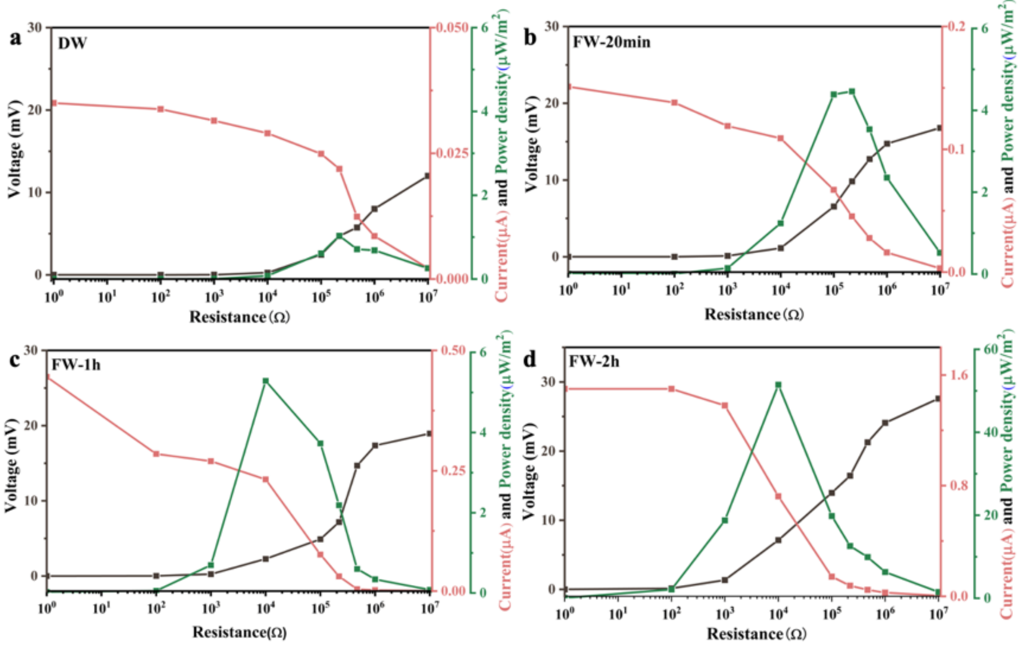

Figure S5. The resistance dependence on voltage, current and power density under 3 sun irradiation of DW (**a**) and FWs with different growth times 20 min (**b**), 1h (**c**), and

2h (**d**).

Table S2. Hydrovoltatic energy harvesting performance from literature

Materials	Medium	Voltage (V)	Current (μA)	Power density* (μW/m^2)	Ref
Nanoengineered wood	Alkaline water (pH=13.4)	1	20	13500	[2]
Nanostructured carbon black	DI water	1	0.15	210	[3]
Porous carbon film	DI water	1	0.600	130	[4]
Al_2O_3/CNT/Epoxy/PMMA	DI water	1.2	0.475	/	[5]
Flexible carbon film	DI water	5	1.5	/	[6]
Porous carbon film	DI water	1.2	0.5	/	[7]
Al_2O_3 film	DI water	2.5	0.8	8	[8]
Porous ZnO film	DI water	0.4	0.02	1	[9]
Ni-Al LDH	DI water	0.7	1.3	120	[10]
Carbon nanosphere/TiO_2 nanowire	DI water	1.6	0.25	/	[11]
Porous carbon	DI water	0.94	0.395	4.8	[12]

film					
Carbonized wood	DI water	0.096	10.5	290	[13]
Fe_3O_4/wood	DI water	1	5.17	742.66	This work

* data in the literature is obtained by calculation based on the device geometry reported.

Table S3. The output performance of FWs

Sample	condition	V_{oc} (V)	I_{sc} (μA)	Power density (μW/m^2)
DW	Without irradiation	0.011	0.06	1.03
	3 sun irradiation	0.105	0.29	8.81
FW-20min	Without irradiation	0.021	0.22	4.46
	3 sun irradiation	0.285	0.87	68.06
FW-1h	Without irradiation	0.03	0.39	5.28
	3 sun irradiation	0.257	1.11	515.85
FW-2h	Without irradiation	0.063	1.17	51.47
	3 sun irradiation	1	5.17	742.66

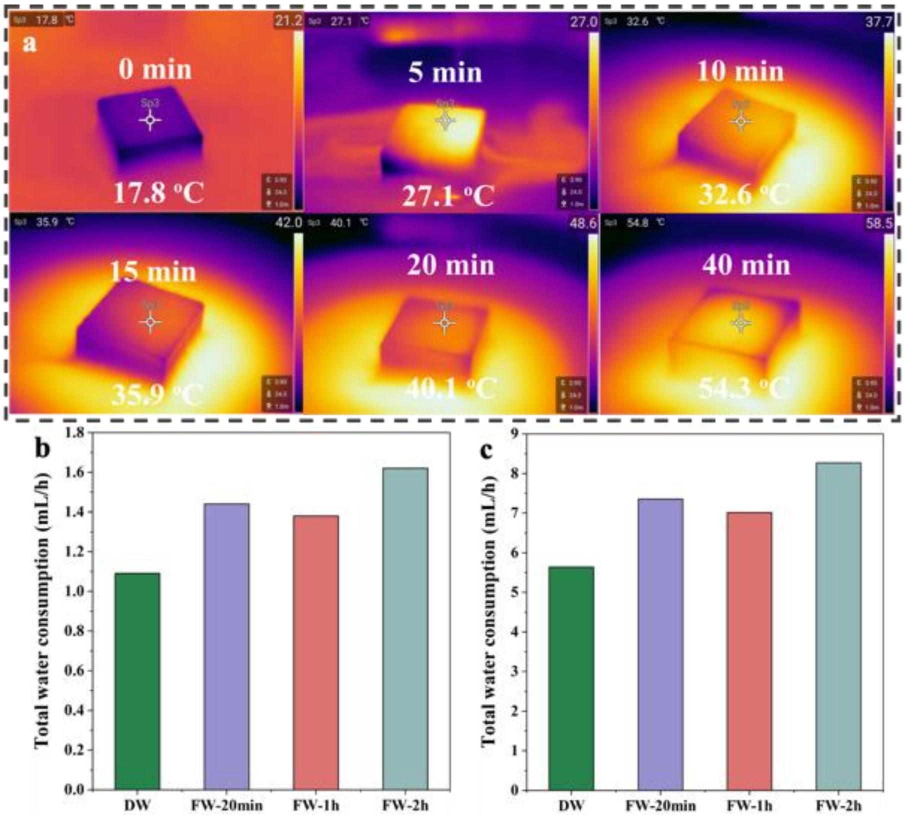

Figure S6. (**a**)Heat localization effect of the DW under 3 sun simulated irradiation. (**b**), (**c**) The rate of total water consumption without irradiation and under 3 sun irradiation.

Table S4. The basic properties FW-10min and FW-4h.

	FW-10min	FW-4h
Zeta potential (mV)	-21.8	-25.1
BET SSA (m^2/g)	2.0	4.7
Fe_3O_4 Content (%)	11.0	23.61
Modulus (MPa)	46.7	21.9

The hydrovoltaic energy harvesters were fabricated by sandwiching the DW or FWs between two Pt mesh electrodes by using the same method with maintext[14]. In order to change the temperature, we put the whole device in the corresponding water bath conditions to ensure that its temperature is not changed, here we tested 40-60 °C.

Solutions with different pH values are prepared by sodium hydroxide, here we tested the conditions with a pH value of 7-13.The open-circuit potential and short circuit current from the wood nanogenerators were recorded in real-time using an Electrochemical workstation (CHI Instruments, model 660E) at 25 °C and 30 % relative humidity. The measurements were carried out by controlling the software on a desktop, and the sampling rate of our instrument was 10 s^{-1}.

Variation in the intensity of the sun will also change the temperature of material/water and evaporation. To study the effect of it, we evaluated the energy harvesting performance of DW and FW-2h at different water temperatures. When water temperature increase from 20 °C to 40 °C, the output voltage is slightly increased due to the enhanced evaporation of water. Further, an increase in the temperature decreases the output voltage, which can be attributed to the increased swelling of wood at higher temperatures. The increased swelling can reduce the porosity at the nanoscale and hence minimize the active surface area for water-material interaction. We evaluated the hydrovoltaic energy harvesting performance of the DW and FW-2h under neutral to alkaline pH conditions as acidic conditions can dissolve the Fe_3O_4. With the increased pH increases the output voltage due to increased surface charge and due to the proton/pH gradient between the top and bottom of the wood, which is consistent with our previous study [2]. However, the wood swells significantly in alkali, which will lead to structural collapse.

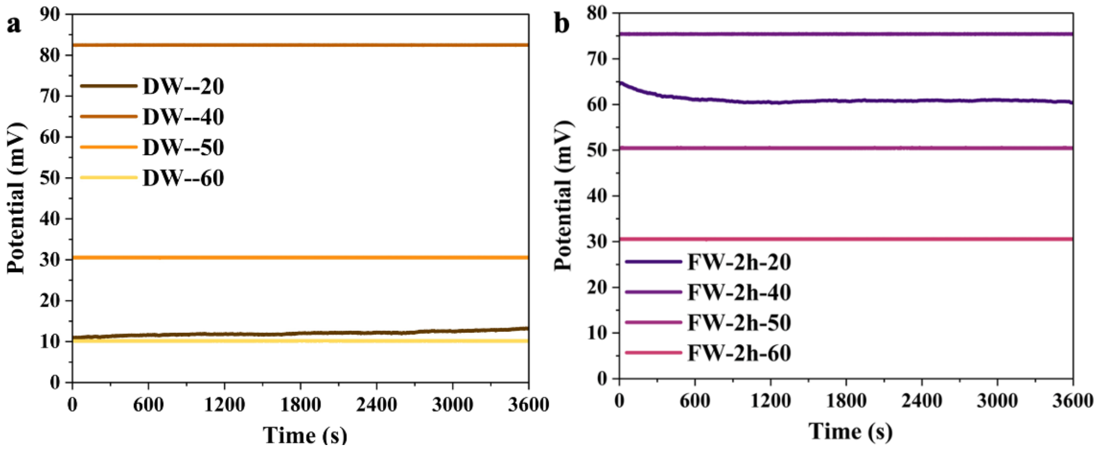

Figure S7. Hydrovoltaic energy harvesting from DW and FW-20 under different

temperature: (**a**) V_{oc} of DW sample; (**b**) V_{oc} of FW-2h sample.

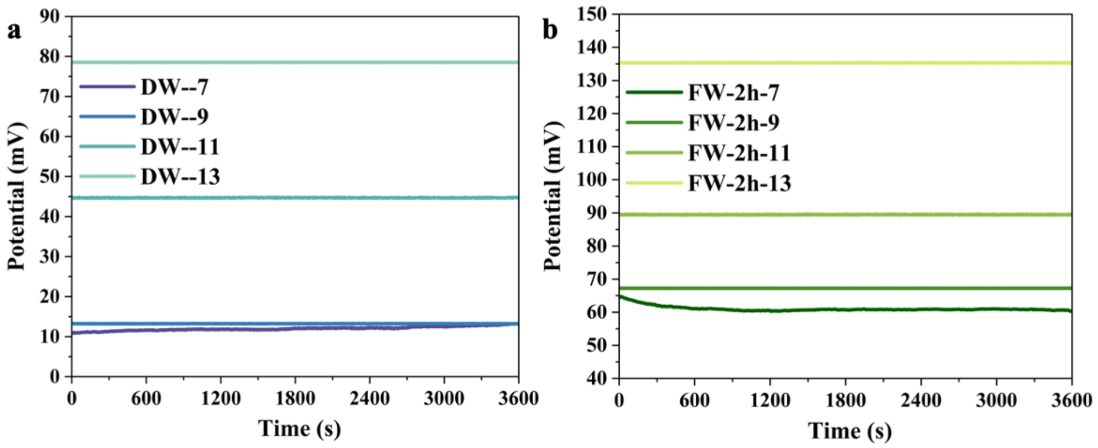

Figure S8. Hydrovoltaic energy harvesting from DW and FW-20 under different pH solution: (**a**) V_{oc} of DW sample; (**b**) V_{oc} of FW-2h sample.

Reference

(1) Gao, Y.; Aliques Tomas, M. del C.; Garemark, J.; Sheng, X.; Berglund, L.; Li, Y. Olive Stone Delignification Toward Efficient Adsorption of Metal Ions. *Front Mater* **2021**, *8*, 605931. https://doi.org/10.3389/fmats.2021.605931.

(2) Garemark, J.; Ram, F.; Liu, L.; Sapouna, I.; Cortes Ruiz, M. F.; Larsson, P. T.; Li, Y. Advancing Hydrovoltaic Energy Harvesting from Wood through Cell Wall Nanoengineering. *Adv Funct Mater* **2023**, *33(4):* 2208933. https://doi.org/10.1002/adfm.202208933.

(3) Xue, G.; Xu, Y.; Ding, T.; Li, J.; Yin, J.; Fei, W.; Cao, Y.; Yu, J.; Yuan, L.; Gong, L.; Chen, J.; Deng, S.; Zhou, J.; Guo, W. Water-Evaporation-Induced Electricity with Nanostructured Carbon Materials. *Nat Nanotechnol* **2017**, *12* (4), 317–321. https://doi.org/10.1038/nnano.2016.300.

(4) Ding, T.; Liu, K.; Li, J.; Xue, G.; Chen, Q.; Huang, L.; Hu, B.; Zhou, J. All-Printed Porous Carbon Film for Electricity Generation from Evaporation-Driven Water Flow. *Adv Funct Mater* **2017**, *27* (22). https://doi.org/10.1002/adfm.201700551.

(5) Zhong, T.; Guan, H.; Dai, Y.; He, H.; Xing, L.; Zhang, Y.; Xue, X. A Self-Powered Flexibly-Arranged Gas Monitoring System with Evaporating Rainwater as Fuel for Building Atmosphere Big Data. *Nano Energy* **2019**, *60*, 52–60. https://doi.org/10.1016/j.nanoen.2019.03.041.

(6) Li, J.; Liu, K.; Ding, T.; Yang, P.; Duan, J.; Zhou, J. Surface Functional Modification Boosts the Output of an Evaporation-Driven Water Flow Nanogenerator. *Nano Energy* **2019**, *58*, 797–802. https://doi.org/10.1016/j.nanoen.2019.02.011.

(7) He, H.; Zhao, T.; Guan, H.; Zhong, T.; Zeng, H.; Xing, L.; Zhang, Y.; Xue, X. A

Water-Evaporation-Induced Self-Charging Hybrid Power Unit for Application in the Internet of Things. *Sci Bull* **2019**, *64* (19), 1409–1417. https://doi.org/10.1016/j.scib.2019.06.020.

(8) Shao, C.; Ji, B.; Xu, T.; Gao, J.; Gao, X.; Xiao, Y.; Zhao, Y.; Chen, N.; Jiang, L.; Qu, L. Large-Scale Production of Flexible, High-Voltage Hydroelectric Films Based on Solid Oxides. *ACS Appl Mater Interfaces* **2019**, *11* (34), 30927–30935. https://doi.org/10.1021/acsami.9b09582.

(9) Yoon, S. G.; Yang, Y. J.; Yoo, J.; Jin, H.; Lee, W. H.; Park, J.; Kim, Y. S. Natural Evaporation-Driven Ionovoltaic Electricity Generation. *ACS Appl Electron Mater* **2019**, *1* (9), 1746–1751. https://doi.org/10.1021/acsaelm.9b00419.

(10) Sun, J.; Li, P.; Qu, J.; Lu, X.; Xie, Y.; Gao, F.; Li, Y.; Gang, M.; Feng, Q.; Liang, H.; Xia, X.; Li, C.; Xu, S.; Bian, J. Electricity Generation from a Ni-Al Layered Double Hydroxide-Based Flexible Generator Driven by Natural Water Evaporation. *Nano Energy* **2019**, *57*, 269–278. https://doi.org/10.1016/j.nanoen.2018.12.042.

(11) Ji, B.; Chen, N.; Shao, C.; Liu, Q.; Gao, J.; Xu, T.; Cheng, H.; Qu, L. Intelligent Multiple-Liquid Evaporation Power Generation Platform Using Distinctive Jaboticaba-like Carbon Nanosphere@TiO2 Nanowires. *J Mater Chem A Mater* **2019**, *7* (12), 6766–6772. https://doi.org/10.1039/c8ta12328d.

(12) Liu, K.; Ding, T.; Li, J.; Chen, Q.; Xue, G.; Yang, P.; Xu, M.; Wang, Z. L.; Zhou, J. Thermal–Electric Nanogenerator Based on the Electrokinetic Effect in Porous Carbon Film. *Adv Energy Mater* **2018**, *8* (13). https://doi.org/10.1002/aenm.201702481.

(13) Zhang, Z.; Zheng, Y.; Jiang, N.; Hong, W.; Liu, T.; Jiang, H.; Hu, Y.; Li, C. Electricity Generation from Water Evaporation through Highly Conductive Carbonized Wood with Abundant Hydroxyls. *Sustain Energy Fuels* **2022**, *6* (9), 2249–2255. https://doi.org/10.1039/d2se00309k.

(14) Zhou, X.; Zhang, W.; Zhang, C.; Tan, Y.; Guo, J.; Sun, Z.; Deng, X. Harvesting Electricity from Water Evaporation through Microchannels of Natural Wood. *ACS Appl Mater Interfaces* **2020**, *12* (9), 11232–11239. https://doi.org/10.1021/acsami.9b23380.
